# Multicenter randomized comparative trial of Micromedex, Micromedex with Watson, or Google to answer drug information questions

**DOI:** 10.5195/jmla.2021.1085

**Published:** 2021-04-01

**Authors:** Christopher Giuliano, Sean McConachie, Julie Kalabalik-Hoganson

**Affiliations:** 1 ek2397@wayne.edu, Associate Professor (Clinical), Wayne State University Eugene Applebaum College of Pharmacy and Health Sciences, Detroit, MI; 2 sean.mcconachie@wayne.edu, Assistant Professor (Clinical), Wayne State University Eugene Applebaum College of Pharmacy and Health Sciences, Detroit, MI; 3 juliek@fdu.edu, Associate Professor and Director of Pharmacy Practice, Fairleigh Dickinson University School of Pharmacy and Health Sciences, Florham Park, NJ

**Keywords:** information storage and retrieval, internet, medical informatics, pharmacy student

## Abstract

**Objective:**

The purpose of this study was to compare pharmacy students’ ability to correctly answer drug information questions using Micromedex with Watson, Micromedex without Watson, or Google.

**Methods:**

This multicenter randomized trial compared pharmacy student responses to drug information questions using Micromedex with Watson, Micromedex without Watson, or Google from January to March of 2020. First- to fourth-year pharmacy students at two institutions were included. The primary outcome was the number of correct answers. Secondary outcomes were the time taken to answer the questions and differences in number of correct answers by pharmacy student year and institution.

**Results:**

The analysis included 162 participants: 52 students in the Micromedex group, 51 students in the Watson group, and 59 students in the Google group. There was a significant difference among groups in the total number of questions answered correctly (p=0.02). Post-hoc analysis revealed that participants in the Micromedex group answered more questions correctly than those in the Google group (p=0.015). There were no significant differences between Micromedex and Watson groups (p=0.52) or between Watson and Google groups (p=0.22). There was also no difference in time to complete the questions among groups (p=0.72).

**Conclusion:**

Utilizing Google did not save students time and led to more incorrect answers. These findings suggest that health care educators and health sciences librarians should further reinforce training on the appropriate use of drug information resources.

## INTRODUCTION

The “Google Generation” has been defined as those born after 1993 and those “with little or no recollection of life before the web” [1]. This generation of “digital natives,” who are familiar and comfortable with technology from a young age, often represent today's students. Although they have matured in an information-rich era, this generation may lack understanding of how information is structured online and desire quick answers, which may contribute to poor search strategies and reliance on general search engines [2]. Search engines like Google are often easy to use; however, their search results may include blogs, anecdotal experiences, outdated information, or basic medical information that is not patient specific. Additionally, the accuracy and reliability of information found through general search engine queries can vary [3].

Micromedex® is a subscription-based clinical decision support system containing information related to medications, disease states, toxicology, and alternative medicine. This database is commonly used by pharmacists and health sciences librarians to teach health care students how to identify reliable and accurate drug information. In 2018, International Business Machines (IBM®) incorporated IBM Watson® into Micromedex. Watson uses natural language processing, hypothesis generation and evaluation, and dynamic learning to provide answers to a stated question. The format for entering questions is similar to a Google search bar, although the information provided is from Micromedex, which should improve its reliability. Providing students with an alternative to Google may help improve clinical decision making. Watson has been evaluated for varying uses in health care research, including decision making surrounding intravenous contrast and cancer treatment [4–6].

Understanding which resources provide the correct answers in the shortest time is important as health care professionals have limited time in daily practice. It has been reported that clinicians spend less than two minutes searching for an answer to a medical question [7, 8]. The desire for quick answers is also observed in health care education. Medical students frequently use electronic resources such as Google without having received formal instruction on medical information retrieval and search strategies [9].

Health care professional trainees may prefer using Google to find health information because of its ease of use, although the reliability of information depends on the source consulted [10]. In a study comparing heuristics versus Google searches, medical residents who used Google correctly diagnosed renal diseases less often than attending physicians [11]. Another study showed that medical students presented with a challenging diagnostic case most commonly used Google. However, more students in the electronic diagnosis support system group identified the correct diagnosis compared with those who used Google or other resources [12]. By contrast, Kim and colleagues compared the speed and accuracy of medical interns in answering clinical questions using Google compared to summary resources and found no significant differences in time to correct response or correct response rate [13]. However, no studies to date have evaluated Micromedex with or without Watson compared to Google in the setting of answering drug information questions. Therefore, the purpose of this study was to compare drug information responses from pharmacy students using Micromedex with Watson, Micromedex without Watson, or Google.

## METHODS

The authors conducted a multicenter randomized controlled trial comparing pharmacy student responses to drug information questions using Micromedex with Watson (Watson), Micromedex without Watson (Micromedex), or Google from January to March of 2020. First- to fourth-year pharmacy students eighteen years of age or older at Wayne State University and Farleigh Dickinson University were included. No exclusion criteria were present. Student responses were not included if they were duplicates or if they did not complete the questionnaire. Institutional review board approval was obtained at both institutions prior to the start of the trial. Course content covering drug information databases and evaluation of websites was taught during the first semester of pharmacy school at Wayne State University and throughout the first and second year at Farleigh Dickinson University.

The primary outcome was the number of correct answers from a series of drug information questions. Secondary outcomes were the time taken to answer the questions across drug information resources and differences in the number of correct answers depending on pharmacy student year and institution.

### Procedures

Students were recruited during a lunch event and via subsequent emails sent at both institutions. Pizza was provided as an incentive, and students were entered into a raffle for 1 of 5 $50 gift cards. Students received an information sheet describing the study prior to starting the questionnaire (supplemental Appendix A). Randomization was both performed electronically and delivered through Qualtrics®. The questionnaire consisted of a total of twenty questions composing ten main constructs delivered through two cases (supplemental Appendix B). Constructs included indication, adult dosing, pediatric dosing, contraindications, black box warning, drug interaction, intravenous compatibility, mechanism of action, monitoring parameters, and storage. Both cases had an identical number of questions, identical formatting with the exception of drug name, and one question per construct. Two cases were used to evaluate consistency of responses. Questions were developed targeting medications not commonly used to decrease the chance that students’ existing knowledge would drive responses. All students received the same questions and were reminded to use the assigned resource while filling out the questionnaire.

Content validation of the questionnaires was performed by three pharmacists and five pharmacy residents. Demographic data collected included age; gender; year in pharmacy school; highest degree obtained; primary language; frequency of use of Google, Micromedex, and Watson; preference ranking for different drug information programs; perceived quality ranking for different drug information programs; and which drug information applications students had installed on their phone.

### Statistical analysis

Sample size was calculated to find a 5% difference in scores between groups with an expected score using Micromedex of 90%, a standard deviation of 13%, an alpha error rate of 0.05, and 80% power. This resulted in a target sample size of 106 students per group. Descriptive statistics were computed using the mean and standard deviation for continuous variables, median and interquartile range for ordinal variables, and frequency distributions for categorical variables. Differences among groups were compared using analysis of variance (ANOVA) and Tukey honestly significant difference (HSD) for post hoc testing or Mann-Whitney U if data were nonparametric. If differences among groups existed and were associated with the outcome (*p*<0.1), linear regression was performed. All data were analyzed using SPSS v. 25.0, and a *p*-value of 0.05 or less was considered to indicate statistical significance.

## RESULTS

A total of 276 students began the questionnaire in the trial period, of which 172 completed all questions. Following deletion of duplicate entries (n=10), 162 responses were included in the final analysis, including 52 students in the Micromedex group, 51 students in the Watson group, and 59 students in the Google group. Full questionnaire completers were not significantly different in baseline demographics compared with non-completers in terms of age (25.3 versus 24.9), proportion of women (69.3% versus 68.2%), or daily use of drug information sources.

**Figure 1:**
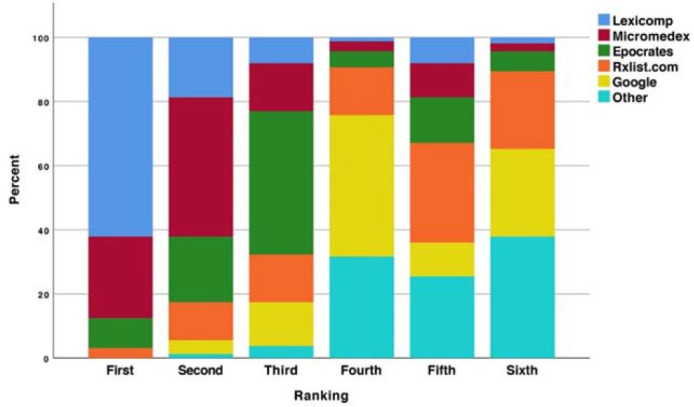
Student ranking for preferred drug information source

**Figure 2:**
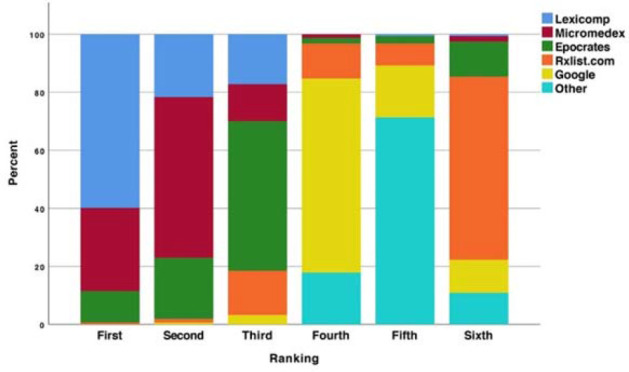
Student ranking of perceived quality of drug information source

The majority of participating students were from Wayne State University (66%), were women (69%), and had obtained a bachelor's degree prior to enrollment in the doctor of pharmacy (PharmD) program (82%). The average age of participants was 25 years old, and the median year in pharmacy school was year 3. In terms of prior experiences, most students were currently employed in the community setting (57%), and the most common career goal was to pursue a residency or fellowship following graduation (37%). The usage of various drug information resources in regular practice varied widely across participants. At baseline, 61% of students indicated they utilized Micromedex at least weekly, 31% of students indicated they used Watson at least weekly, and 81% of students indicated they used Google for drug information at least weekly. The majority of students ranked Lexicomp® as their most-preferred drug information database (62%), followed by Micromedex (25%) (Figure 1). Similar results were identified with regard to perceived quality of drug information resources (Figure 2). Specifically, Lexicomp and Micromedex were perceived as the highest quality resources (58% and 28%, respectively). Epocrates was most commonly ranked as the lowest quality drug information resource (61%).

The 3 treatment groups were well balanced in terms of demographic characteristics, with the exception of the frequency of baseline Micromedex usage and randomization to a database that was used at least weekly by the students (Table 1). However, neither baseline Micromedex usage (F(5,156)=2.05, *p=*0.074) nor matching drug information database (F(1,160)=0.59, *p=*0.44) were associated with the primary outcome.

**Table 1:** Baseline characteristics^*^

Group	Micromedex (n=52)		Watson (n=51)		Google (n=59)		Statistical significance	
	Mean	(SD)	Mean	(SD)	Mean	(SD)		
Age:	25.5	(± 3.3)	25.3	(± 3.6)	25.1	(± 3.2)	F(2,159)=0.22	*p*=0.81
	n	(%)	n	(%)	n	(%)		
Pharmacy school							χ^2^(2)=0.23	*p*=0.89
Wayne State	34	(65%)	35	(69%)	38	(64%)		
Fairleigh Dickinson	18	(35%)	16	(31%)	21	(36%)		
Year in school							χ^2^(6)=2.22	*p*=0.90
First	10	(19%)	9	(18%)	10	(17%)		
Second	13	(25%)	12	(24%)	15	(25%)		
Third	18	(35%)	6	(12%)	24	(41%)		
Fourth	11	(21%)	14	(27%)	10	(17%)		
Female sex	37	(71%)	35	(69%)	40	(68%)	χ^2^(2)=0.16	*p*=0.93
Highest degree obtained							χ^2^(6)=6.10	*p*=0.41
High school diploma	1	(2%)	2	(4%)	4	(7%)		
Associate's degree	3	(6%)	2	(4%)	8	(14%)		
Bachelor's degree	45	(87%)	43	(84%)	44	(75%)		
Master's degree or higher	4	(8%)	4	(8%)	3	(5%)		
Current employment							χ^2^(6)=9.67	*p*=0.14
Community	31	(60%)	22	(43%)	39	(66%)		
Hospital or ambulatory care	12	(23%)	11	(22%)	10	(17%)		
Other pharmacy	2	(4%)	8	(16%)	5	(8%)		
Nonpharmacy or unemployed	7	(13%)	10	(20%)	5	(8%)		
Career plans							χ^2^(8)=7.38	*p*=0.50
Community	15	(29%)	8	(16%)	18	(31%)		
Hospital or ambulatory care	6	(12%)	8	(16%)	8	(14%)		
Residency/fellowship	17	(33%)	22	(43%)	21	(36%)		
Other	7	(13%)	10	(20%)	9	(15%)		
Undecided	7	(13%)	3	(6%)	3	(5%)		
Drug references on computer							χ^2^(2)=4.83	*p*=0.09
Single reference	0	(—)	2	(4%)	5	(8%)		
Multiple references	52	(100%)	49	(96%)	54	(92%)		
Mobile references							χ^2^(10)=4.83	*p*=0.90
Lexicomp	7	(13%)	5	(10%)	10	(17%)		
Epocrates	4	(8%)	4	(8%)	5	(8%)		
Google	5	(10%)	10	(20%)	10	(17%)		
Other	6	(12%)	3	(6%)	4	(7%)		
Multiple	26	(50%)	24	(47%)	24	(41%)		
None	4	(8%)	5	(10%)	6	(10%)		
Computer database match							χ^2^(2)=46.9	*p*<0.01
Yes	13	(25%)	41	(80%)	13	(22%)		
No	39	(75%)	10	(20%)	46	(78%)		
Mobile database match							χ2(2)=3.27	p=0.20
Yes	37	(71%)	38	(75%)	35	(59%)		
No	15	(29%)	13	(25%)	24	(41%)		
Baseline Micromedex usage							H(2)=8.52	p=0.01
Multiple times per day	7	(13%)	3	(6%)	1	(2%)		
Daily	5	(10%)	2	(4%)	3	(5%)		
Several times per week	17	(33%)	15	(29%)	16	(27%)		
Weekly	10	(19%)	7	(14%)	13	(22%)		
Monthly	6	(12%)	8	(16%)	15	(25%)		
Never	7	(13%)	16	(31%)	11	(19%)		
Baseline Watson usage							H(2)=3.81	*p*=0.15
Multiple times per day	1	(2%)	2	(4%)	1	(2%)		
Daily	2	(4%)	2	(4%)	0	(—)		
Several times per week	7	(13%)	6	(12%)	14	(24%)		
Weekly	7	(13%)	0	(—)	9	(15%)		
Monthly	5	(10%)	8	(16%)	9	(15%)		
Never	30	(58%)	33	(65%)	26	(44%)		
Baseline Google usage							H(2)=4.57	*p*=0.10
Multiple times per day	15	(29%)	8	(16%)	12	(20%)		
Daily	9	(17%)	13	(25%)	9	(15%)		
Several times per week	17	(33%)	8	(16%)	16	(27%)		
Weekly	6	(12%)	9	(18%)	9	(15%)		
Monthly	3	(6%)	7	(14%)	9	(15%)		
Never	2	(4%)	6	(12%)	4	(7%)		

* Percentages may add up to more than 100% due to rounding to nearest 1%.

In terms of the primary outcome of the total number of correct answers across both cases, there was a significant difference among groups in the total number of questions answered correctly (Table 2). Post-hoc analysis revealed that participants in the Micromedex group answered significantly more questions correctly than those in the Google group (mean difference=2.18; *p=*0.015). However, there was no difference between Micromedex and Watson groups (mean difference=0.88; *p=*0.52) or between Watson and Google groups (mean difference=1.31; *p=*0.22). Adjusting for baseline Micromedex use did not change the difference observed in the primary outcome.

**Table 2:** Outcomes

Group	Micromedex (n=52)		Watson (n=51)		Google (n=59)		Statistical significance	
	Mean	(SD)	Mean	(SD)	Mean	(SD)		
Total correct answers:	18.3	(± 3.42)	17.4	(± 4.45)	16.1	(± 4.16)	F(2,159)=4.05	*p*=0.02
Total duration in minutes	42.0	(± 63.3)	40.3	(± 58.3)	85.9	(± 56.2)	F(2,159)=0.32	*p*=0.72
Total correct answers (case 1)	9.2	(± 1.66)	8.9	(± 2.00)	8.6	(± 2.06)	F(2,159)=1.31	*p*=0.27
Total correct answers (case 2)	9.1	(± 2.01)	8.5	(± 2.64)	7.5	(± 2.49)	F(2,159)=6.11	*p*<0.01

These results were driven primarily by differences in correct responses from case 2. For case 1, there was no difference in the number of correct answers among groups despite both cases utilizing similar question types. In terms of the time required to complete the questionnaire, there was no difference among groups; therefore, no post-hoc analyses were conducted.

There was no difference in total number of correct answers depending on institution (*p*=0.78). However, there was a significant difference in performance by student year (F(3,158)=4.22, *p=*0.007), with the number of correct answers tending to be higher for third- and fourth-year students compared to first-year students (mean difference: 2.96, *p=*0.008 and 2.41, *p=*0.07, respectively). Lastly, sensitivity analysis was conducted by excluding participants who completed the entire questionnaire in under 5 minutes (n=8). This exclusion did not result in any significant changes to the comparative baseline analyses between groups, primary outcome analysis, or need for linear regression analysis.

## DISCUSSION

This is the first study to compare the ability of students to answer drug information questions correctly depending on whether they used Micromedex with or without Watson or Google. We found that students using Micromedex performed better than those using Google, whereas there was no difference in performance between students using Watson versus Micromedex. Differences were primarily driven by case 2, although both cases used the same constructs and formatting, with the only difference being drug name. It is possible that the Google results for the drugs in case 2 questions were more difficult to locate or less accurate. Reviewing these results, it seems like it should be obvious that students should use a trusted drug information sources rather than Google, as this is taught to students both in the classroom and on clinical rotations. However, the majority of students admitted to using Google as a drug information resource at least weekly, which was similar to what we have observed in practice. Although time to complete the questionnaire did not differ among groups, this was mainly a result of the large variance in time it took to answer the questions. We hypothesize that this large variance was a result of the amount of the time it took for students to choose a website from the Google search results, become familiar with the chosen website, evaluate the website after arriving at it, and find the information on the website.

Similar findings have been observed in other research with health care professionals. In a web-based survey of medical students, Google was one of the most commonly used electronic resources; however, use of Google resulted in students correctly answering only 18% of medical queries [14]. Additionally, medical students who were presented with a challenging diagnostic case were less likely to choose the correct diagnosis when utilizing Google [12]. Results might differ with more clinical training, as Kim and colleagues found no significant difference in mean time to find the correct response or mean correct response rate for medical interns [13]. Of note, we observed higher overall scores for students in their final two years of pharmacy school, which suggested that students improved their drug information interpretation over time, despite the resource utilized.

Our findings have important implications for health sciences librarians and pharmacists. We should continue to reinforce that Google will not save students time in answering drug information questions and the answers that they find are less likely to be correct. Having evidence to reinforce this point is important. It is possible that this trial could be recreated as an educational activity in the classroom. Students could look up information in small groups, with each student assigned to different resources. After students find their answers, they could discuss how the information they found varies. Of note, we chose rare medications, so our results might not be the same as an evaluation of commonly used medications, as preexisting knowledge may obscure any differences among resources.

Our study had some strengths and limitations. Randomization helped to create similar characteristics amongst groups, our study utilized more than one pharmacy school, and our questions underwent content validation. However, our study did not have full student body participation, which may have influenced our findings. Additionally, a significant number of students only completed the baseline demographics component of the questionnaire. Once arriving at the drug information questions, many students abandoned the questionnaire because they felt the questions were too difficult since they had not previously encountered the medications. This was ascertained from student feedback to us. However, students who only filled out the demographic questionnaire were similar in age, gender, and drug information database preference, making this limitation less likely to influence our results. Lastly, we did not meet our sample size, although we were able to demonstrate a significant difference in our primary outcome.

## CONCLUSION

Pharmacy students who used Micromedex answered more drug information questions correctly compared to those who used Google, although the time to answer questions did not differ among groups. Google was the most frequently used resource used by students outside of the trial despite its perceived low quality. These findings suggested that health care educators and health sciences librarians should provide students access to reliable drug information and further reinforce training on the appropriate use of drug information resources.

## Data Availability

Data associated with this article are available at Figshare at https://figshare.com/articles/dataset/Drug_Information_Database_Trial/13061165.

## SUPPORTING AGENCIES

American College of Clinical Pharmacy Education and Training Practice and Research Network.

## SUPPLEMENTAL FILES

**Appendix A:** Research information sheet

**Appendix B:** Drug information cases
