# Supplementary material for: Multicenter randomized comparative trial of Micromedex, Micromedex with Watson, or Google to answer drug information questions
Source: J Med Libr Assoc. 2021 Apr 1;109(2):212–8. doi: 10.5195/jmla.2021.1085 (PMC8270367; doi:10.5195/jmla.2021.1085)
Supplement: Supplementary file 1 — Appendix A: Research information sheet [file jmla-109-2-212-s01.docx]

# Multicenter randomized comparative trial of Micromedex, Micromedex with Watson, or Google to answer drug information questions

## Christopher Giuliano; Sean McConachie; Julie Kalabalik-Hoganson

### APPENDIX A

### Research information sheet

Principal Investigator (PI): Christopher Giuliano, PharmD, MPH, Pharmacy Practice

**Purpose:**

You are being asked to be in a research study evaluating use of drug information resources because you are a pharmacy student. This study is being conducted at Wayne State University Eugene Applebaum College of Pharmacy and Health Sciences and Farleigh Dickinson University School of Pharmacy and Health Sciences.

**Study procedures:**

If you take part in the study, you will be asked to:

- Take a survey using a drug information resource to answer a clinical case. The survey is expected to take thirty minutes.
- This survey is not part of a course and will not affect any grades.
- You will have to complete all questions in order to have your answers recorded and to remain in the survey. A link to a second survey will open after the survey’s completion for the opportunity to win a $50 Amazon gift card. Food will be provided while taking the survey.

**Benefits:**

- As a participant in this research study, there will be no direct benefit for you; however, information from this study may benefit other people now or in the future. Food will be provided while taking the survey.

**Risks:**

- There are no known risks at this time to participation in this study.

**Costs:**

- There will be no costs to you for participation in this research study.

**Compensation:**

- You will not be paid for taking part in this study. However, upon completion of the survey, you will be provided with an additional link into a second Qualtrics® survey to be entered into a drawing to win 1 of 5 $50 Amazon gift cards.

**Confidentiality:**

- All information collected about you during the course of this study will be kept without any identifiers.
- You will be identified in the research records by a code name or number.

**Voluntary participation/withdrawal:**

Taking part in this study is voluntary. You are free to not answer any questions or withdraw at any time. Your decision will not change any present or future relationships with Wayne State University or Farleigh Dickinson University or its affiliates.

**Questions:**

If you have any questions about this study now or in the future, you may contact Christopher Giuliano or one of the research team members, Julie Kalabalik. If you have questions or concerns about your rights as a research participant, the chair of the institutional review board can be contacted. If you are unable to contact the research staff, or if you want to talk to someone other than the research staff, you may also call the Wayne State research subject advocate to discuss problems, obtain information, or offer input.

**Participation:**

By completing the survey, you are agreeing to participate in this study. The data that you provide may be collected and used by Qualtrics as per its privacy agreement.
