# Supplementary material for: Multicenter randomized comparative trial of Micromedex, Micromedex with Watson, or Google to answer drug information questions
Source: J Med Libr Assoc. 2021 Apr 1;109(2):212–8. doi: 10.5195/jmla.2021.1085 (PMC8270367; doi:10.5195/jmla.2021.1085)
Supplement: Supplementary file 2 — Appendix B: Drug information cases [file jmla-109-2-212-s02.docx]

# Multicenter randomized comparative trial of Micromedex, Micromedex with Watson, or Google to answer drug information questions

## Christopher Giuliano; Sean McConachie; Julie Kalabalik-Hoganson

### APPENDIX B

### Drug information cases

| **Construct** | **Case 1** | **Case 2** |
| --- | --- | --- |
| Indication | A patient presents to your pharmacy and states she saw a commercial on TV for a drug called Dupixent®. She would like to know what disease this drug is used to treat so that she can ask her doctor if it is a possible treatment option for her condition. What is the indication of this drug?   - Plaque psoriasis - Chronic hepatitis C virus - Refractory acute myeloid leukemia - Atopic dermatitis* | A patient presents to your pharmacy and states she saw a commercial on TV for a drug called Siliq®. She would like to know what disease this drug is used to treat so that she can ask her doctor if it is a possible treatment option for her condition. What is the indication of this drug?   - Acne vulgaris - Plaque psoriasis* - Venous thromboembolism - Refractory acute lymphoblastic leukemia |
| Adult dosing | Your Advance Pharmacy Practice Experience (APPE) preceptor asks you about a newly Food and Drug Administration (FDA)–approved drug called safinamide. He would like to know what the appropriate initial dosing is for an adult patient with Parkinson’s disease who has normal renal and hepatic function. What is the correct initial dosing?   - 25 milligrams (mg) - 50 mg* - 75 mg - 100 mg | Your APPE preceptor asks you about a newly FDA approved drug called sarilumab. He would like to know what the appropriate initial dosing is for an adult patient with rheumatoid arthritis who has normal renal and hepatic function. What is the correct initial dosing?   - 50 mg - 100 mg - 150 mg - 200 mg* |
| Pediatric dosing | A woman presents to the clinic with her 14-year-old adolescent. Your APPE preceptor asks about the appropriate initial dosing for benralizumab for this patient. What is the correct initial dosing?   - 15 mg - 30 mg* - 45 mg - 60 mg | A woman presents to the clinic with her 8-year-old child (weight 28 kilograms). Your APPE preceptor asks about the appropriate initial dosing for benznidazole for this patient. What is the correct initial dosing?   - 50 mg every 12 hours - 62.5 mg every 12 hours - 75 mg every 12 hours* - 100 mg every 12 hours |
| Contraindication | A physician calls your pharmacy asking about a newly approved drug called Steglatro®. She would like to know if she can initiate this drug for her patient and recalls that certain patients should not receive the drug. The physician asks for the drug contraindications. Which of the following is a contraindication?   - Severe hepatic impairment - End stage renal disease* - Hypersensitivity to insulin - Age less than eighteen years | A physician calls your pharmacy asking about a newly approved drug called Trulance®. She would like to know if she can initiate this drug for her patient and recalls that certain patients should not receive the drug. The physician asks for the drug contraindications. Which of the following is a contraindication?   - Age less than eighteen years - Mechanical gastrointestinal obstruction* - Mild hepatic impairment - Hemodialysis |
| Black box warning | Your professor is discussing a newly approved drug called plecanatide. Your professor mentions this drug has a black box warning and asks you to describe the black box warning to the class. What is the correct black box warning?   - Age less than 6 years* - Creatinine clearance less than 60 mL/min - Stevens-Johnson syndrome - Hypersensitivity reaction in HLA-B*5701 allele carriers | Your professor is discussing a newly approved drug called Austedo®. Your professor mentions this drug has a black box warning and asks you to describe the black box warning to the class. What is the correct black box warning?   - Depression and suicidality* - Age less than eighteen years - Severe renal impairment - Concomitant use of anticholinergic drugs |
| Drug interaction | A patient is being discharged from the hospital on betrixaban. He is going to be restarted on his medication for depression (escitalopram). As the transitional care pharmacist, you are asked to review the two drugs for any possible drug-drug interactions. Which of the following is true?   - There is no drug-drug interaction between betrixaban and escitalopram. - Concurrent use of betrixaban and escitalopram may increase risk of bleeding.* - Concurrent use of betrixaban and escitalopram may increase risk of clotting. - A drug-drug interaction is only present in patients with poor renal function. | A patient is being discharged from the hospital on delafloxacin. He is going to be restarted on his iron supplement. As the transitional care pharmacist, you are asked to review the two drugs for any possible drug-drug interactions. Which of the following is true?   - There is no drug-drug interaction between delofloxacin and iron. - Concurrent use of delafloxacin and iron may result in decreased delafloxacin efficacy.* - Concurrent use of delafloxacin and iron may result in increased delafloxacin efficacy. - This drug-drug interaction is only present in patients with poor hepatic function. |
| Intravenous (IV) compatibility | You are asked to present a drug monograph for Giapreza® at your hospital’s pharmacy and therapeutics committee meeting for possible formulary addition. Following your presentation, the nurse manager asks about the IV compatibility with this drug, specifically if it is compatible with normal saline (0.9% sodium chloride). Which of the following is the correct response?   - Giapreza® is compatible with normal saline (0.9% sodium chloride).* - Giapreza® is incompatible with normal saline (0.9% sodium chloride). | You are asked to present a drug monograph for Besponsa® at your hospital’s pharmacy and therapeutics committee meeting for possible formulary addition. Following your presentation, the nurse manager asks about the IV compatibility with this drug, specifically if it is compatible with normal saline (0.9% sodium chloride). Which of the following is the correct response?   - Besponsa® is compatible with normal saline (0.9% sodium chloride).* - Besponsa® is incompatible with normal saline (0.9% sodium chloride). |
| Mechanism of action | Your APPE preceptor asks you about a newly approved drug called valbenazine. Your preceptor would like you to describe the mechanism of action of this drug. What is the correct mechanism of action?   - Inhibits vesicular monoamine transporter 2* - Synthetic human peptide hormone - CD22-directed antibody-drug conjugate - Inhibits type-B monoamine oxidase | Your APPE preceptor asks you about a newly approved drug called neratinib. Your preceptor would like you to describe the mechanism of action of this drug. What is the correct mechanism of action?   - Inhibits vesicular monoamine transporter 2 - Inhibits epidermal growth factor receptor* - Human monoclonal IgG2 antibody - Human monoclonal IgG1 lambda antibody |
| Monitoring parameters | A patient at your clinic is being initiated on Symproic®. In speaking with the patient, you would like to counsel the patient on what the health care team will be monitoring while he is on this drug. Which of the following are appropriate monitoring parameters for Symproic?   - Frequency of spontaneous bowel movements* - Tuberculosis reactivation - Worsening heart failure - Reductions in acne lesions | A patient at your clinic is being initiated on Mavyret®. In speaking with the patient, you would like to counsel the patient on what the health care team will be monitoring while he is on this drug. Which of the following are the appropriate monitoring parameters for Mavyret?   - Clinical improvement of hepatitis C virus* - Increase in blood pressure - Hemoglobin levels - Blood glucose levels |
| Storage | As a pharmacy technician, you just received a medication shipment to your pharmacy including the drug Rhopressa®. The pharmacist asks you to find out how the drug should be stored, prior to opening. Which of the following is correct regarding storage?   - Store between 2 and 8 degrees C.* - Store between 8 to 15 degrees C. - Store between 15 and 25 degrees C. - Store between 25 and 35 degrees C. | As a pharmacy technician, you just received a medication shipment to your pharmacy including the drug guselkumab. The pharmacist asks you to find out how the drug should be stored, prior to opening. Which of the following is correct regarding storage?   - Store between 2 and 8 degrees C.* - Store between 8 to 15 degrees C. - Store between 15 and 25 degrees C. - Store between 25 and 35 degrees C. |

*=Correct answer.
